# E-health: potential benefits and challenges in providing and accessing sexual health services

**DOI:** 10.1186/1471-2458-13-790

**Published:** 2013-08-30

**Authors:** Victor Minichiello, Saifur Rahman, Tinashe Dune, John Scott, Gary Dowsett

**Affiliations:** 1 The Australian Research Centre in Sex, Health and Society, School of Public Health & Human Biosciences, La Trobe University, Melbourne, Australia; 2 Collaborative Research Network for Mental Health and Well-being, University of New England, Armidale, New South Wales, Australia; 3 School of Behavioural, Cognitive and Social Sciences, University of New England, Armidale, New South Wales, Australia; 4 Australian Research Centre in Sex, Health and Society, La Trobe University, Melbourne, Victoria, Australia

**Keywords:** E-health, Sexual health, Internet, Health service, Access to services

## Abstract

**Background:**

E-health has become a burgeoning field in which health professionals and health consumers create and seek information. E-health refers to internet-based health care and information delivery and seeks to improve health service locally, regionally and worldwide. E-sexual health presents new opportunities to provide online sexual health services irrespective of gender, age, sexual orientation and location.

**Discussion:**

The paper used the dimensions of the RE-AIM model (reach, efficacy, adoption, implementation and maintenance) as a guiding principle to discuss potentials of E-health in providing and accessing sexual health services. There are important issues in relation to utilising and providing online sexual health services. For healthcare providers, e-health can act as an opportunity to enhance their clients’ sexual health care by facilitating communication with full privacy and confidentiality, reducing administrative costs and improving efficiency and flexibility as well as market sexual health services and products. Sexual health is one of the common health topics which both younger and older people explore on the internet and they increasingly prefer sexual health education to be interactive, non-discriminate and anonymous. This commentary presents and discusses the benefits of e-sexual health and provides recommendations towards addressing some of the emerging challenges.

**Future directions:**

The provision of sexual health services can be enhanced through E-health technology. Doing this can empower consumers to engage with information technology to enhance their sexual health knowledge and quality of life and address some of the stigma associated with diversity in sexualities and sexual health experiences. In addition, e-sexual health may better support and enhance the relationship between consumers and their health care providers across different locations. However, a systematic and focused approach to research and the application of findings in policy and practice is required to ensure that E-health benefits all population groups and the information is current and clinically valid and effective, including preventative approaches for various client groups with diverse needs.

## Background

E-health can make a valuable contribution to optimising health across the lifespan and across the globe. According to the South African social rights activist and Archbishop Emeritus, Desmond Tutu, “*e-health is a ray of light on the horizon for the health and equity challenges that plague humanity”*[1]*.* E-health refers to internet-based health care delivery characterized by the movement away from tele-medicine and tele-health. Using the internet and related technologies, e-health seeks to improve health services locally, regionally and worldwide [2]. It is used within the health sector for clinical, educational, preventative, research and administrative purposes, both on-site and remotely. E-health services encompass “six C’s”: content, connectivity, commerce, community, clinical care and computer applications [3,4].

The explosion of technology presents new opportunities to provide online sexual health services irrespective of gender, age, sexual orientation and location. For instance, e-sexual health resources are now accessible from desktop computers to mobile devices and provide e-learning tools to electronic health records, which relate to the management of sexual health problems. Given that the internet has also become a key feature in social life, allowing people to form new relationships, share photos, watch television and seek a plethora of information [5], e-sexual health has the potential to expand its reach from the novel and, often, exclusive to the everyday.

Currently, sexual health related interventions have increasingly turned towards: internet-based sex education; STI screening, testing and management including partner notification through health education websites; online counselling; and social networking and support groups where sexual health services are integrated with other health services [6-9].

Furthermore, the internet, for some social groups, is the preferred outlet for consumers to learn about sexual health, thereby reducing the pressure to talk about sexual health matters with peers, educators, partners and/or healthcare professionals [10]. Young people are especially interested in sexual health information online [11] and search for such information more frequently than older age groups [12]. Their searches include sexualities, body changes, menstruation, physical/sexual abuse, contraception, pregnancy, and sexually transmissible infections (STIs) [13]. However, there are important issues in relation to utilising online sexual health services that impact on their effectiveness.

The aim of this article is to discuss e-sexual health issues including the benefits, drawbacks, challenges and to recommend ways of addressing emerging issues in future research and practice. This article is based on a narrative review of issues related to online sexual health services from the perspectives of providers and consumers. In identifying and presenting relevant information for discussing these issues, the paper adopted the RE-AIM model to assess and analyse the available information under the five RE-AIM recommended assessment dimensions: reach, efficacy (both positive and negative), adoption, implementation and maintenance [14,15].

The paper mostly considered selected peer-reviewed literature and some online resources to analyse the current status of e-sexual health and its impact on sexual health providers and consumers under the five RE-AIM recommended dimensions. The peer-reviewed literature and web-based resources were searched using PubMed and Google respectively. The key words used in search included “e-health”, “e-sexual health” and “e-sexual health services”. Searching PubMed using the key word “e-health” produced 887 unique results. However, adding “sexual health” to “e-health” resulted in the exclusion of the majority of the 887 publications. This paper only considered those publications and web information that focused on e-sexual health and related services. E-health is a relatively recent term in the era of electronically supported healthcare practice, dating back to at least 1999. As such, this narrative review considered the literatures published in or after 1999.

## Reach

This first dimension of e-sexual health investigates its accessibility to a number of populations including adolescents, adults and seniors irrespective of location (i.e., urban versus rural). Worldwide several internet-based sexual health services pilot programs targeting various populations including youth, adults, and gay, lesbian, transgender, intersex and queer individuals with promising results [16,17]. For instance, a study by Noar et al. has shown that computer-based technologies significantly increased condom use and reduced risky sexual behaviour, incidence of STIs and number of sexual partners. This study highlights how computer interventions were successful in reaching a number of population groups with diverse sexual orientations [17]. Surveys consistently show that of the 2.4 billion internet users in 2012 [18], 60-80% of these users have used the internet to obtain health information [19,20]. Further, two thirds of the consumers seeking health information online reported that internet-mediated information has had some impact on their health. In accessing and using information, consumers value anonymity, convenience and quantity of information [21]. Currently, there are approximately 100,000 e-health websites with many providing sexual health services. However, statistics that specifically indicate the effectiveness and reach of e-sexual health websites are rare. Nevertheless, e-sexual health services can be an effective means of removing the barriers of time and distance toward better access to sexual health information and management with full confidentiality, privacy and flexibility around the world.

## Efficacy

It is important that an analysis of e-sexual health includes a discussion of its benefits and drawbacks.

### Benefits

We are living in the age of digital connectivity where the key stakeholders in the e-health industry include providers and consumers [22]. Information technology and consumerism together act as synergetic forces to promote an “information age health system”, where consumers access and utilize health care resources more efficiently [23]. This is particularly relevant to the provision of sexual health care in which consumer privacy and confidentiality are paramount—one of the internet’s greatest strengths [24]. The internet provides information on health and health services and supports self-help and patient choice with the potential to educate and empower the health consumers [25]. As such, the internet can play a vital role in supporting sexual health services provided by health professionals.

For lay persons, the internet becomes a marketplace for general sexual health aids (i.e., condoms, adult toys and lubricants), educational resources, social networking sites (SNS), internet relay chat, e-mail communication, text messaging and blogging. Gold et al. have reported that text messaging to young people significantly increased their knowledge on sexual health and STI testing [26]. Carpenter et al. have reported that a web-based cognitive behavioural skills training and motivational enhancement effectively reduced sexual risk in men who have sex with men (MSM) [16]. Most of the participants in a study conducted by Shoveller et al. indicated that online STI testing and risk assessment provides convenience of circumventing clinic visits, the ability to test privately and instantly, increased privacy and decreased anxiety [9].

### Drawbacks

The potential drawbacks around e-sexual health include difficulty in engaging target groups, eliminating health disparities, communication inequalities and assuring quality of information. Cline and Haynes identified a number of issues for people seeking online health information or knowledge, which include: access difficulty; information overload; disorganisation; search difficulties; overly technical language; lack of user-friendliness; lack of permanence; lack of peer review or regulation; inaccurate, misleading and dangerous information; and maladaptive behaviour [27]. Many of these factors can act as barriers for any internet based sexual health promotion interventions to be effective and have the effect of the health information seeker to be less engaged with such interventions. To be effective, such web based interventions need to be consistently available and attractive enough to keep people engaging and interacting as long as necessary to improve knowledge, or to change attitudes or behaviours.

However, the challenges are how can the websites and the online resource materials of such interventions reach their target audience consistently and over time, and seek the active engagement of the users. There is an urgent research agenda that requires further investigation: for example, what communication strategies are needed; what pedagogical decisions are required in designing these virtual sexual health information centres to facilitate not only high volume of traffic from consumers, but regular site visits over long periods of time and ongoing confidence in using such sites; and finally, what will allow users to bookmark and regularly visit these websites. According to the Health e-Technologies Initiative, an engaging e-health intervention need identification of audience, activation of people to use e-health tools, cultural and linguistic competency/design, consideration of literacy challanges, introduction of technology to unfamiliar audience including addressing security fear, and determination of the quality of information including currency, accuracy, organization, readability and intelligibility [28].

Online sexual health services may face challenges in engaging target groups by meeting the broad range of sexual health needs due to literacy levels, cultural and language differences, age differences, educational differences, and access to technology. The Taiwan Network Information Centre’s survey (2007) reported that people aged 56 and over, on low income, and lacking high school qualifications were less likely to access the internet [29]. As such, some of the most vulnerable groups may benefit the least from e-health. Most e-health websites are designed for individuals with average literacy levels [30]. Multilingualism is an increasing challenge in the utilisation of health services that aim to be universal [31]. In addition, culturally specific mores and norms that affect the acceptability and use of e-health services [30] can act as a challenge particularly for the services related to sexual health.

E-health has significant challenges in eliminating health disparities. Sexual health related services in e-health could primarily serve those with greater resources (i.e., better resourced and integrated health care systems and infrastructure), which can exacerbate health disparities in population subgroups [31]. It is evident that communication inequalities exist when web-based services are utilized by higher-income, highly educated, younger, and employed groups. With the focus of developing and improving technologies to provide effective sexual health services, reaching and accessing various socio-economic and cultural groups is equally an important consideration [32].

The greatest challenge for e-sexual health may be accuracy and reliability of information. According to Maloney and colleagues, difficulty in finding accurate and reliable health information act as a major challenge for e-health [33]. The consequences of inaccurate and incomplete information can be detrimental for consumers and lead to erroneous decision-making [34]. Sexual health related decision-making is sensitive and often vital. While the internet offers a large amount of information on sexual health, many of its contributors and authors (particularly those posting in blogs, SNS and chat-rooms) may not be trained and sufficiently qualified to provide accurate and comprehensive information. Further, sexual health information may be provided with the intention to sell products or services (i.e., erectile dysfunction and premature ejaculation medications and services) instead of provide education. In response to these challenges, initiatives like *Health on the Net* (HON) code and *Health Internet Ethics* (HI-ethics) aim to improve the quality of health information on the internet [35] by creating standards regarding how and what sort of information is presented, how it is organised for consumption, and who acts as ‘educators or advisors’ with consumers.

## Adoption

In light of the potential benefits and drawbacks of e-sexual health its acceptance and adoption by providers and consumers is an important consideration. In the health sector, research indicates that health professionals view e-health as an opportunity to enhance patient care by facilitating communication, reducing administrative costs, improving efficiency and flexibility as well as a way to market health services and products [36]. Additionally, e-health helps to reduce the gap between what is known and what is done in health—the “know-do gap”. In addition, e-health allows health professionals to gather as well as provide high quality information in real-time and enhances their ability to function optimally without the support of information management staff [37]. Further, global information and communications technology networks help practitioners gain access to current and relevant knowledge worldwide, instantly and at low cost in order to contribute effectively to improving people’s health through research and best-practice [37].

The adoption of e-sexual health services can also be of benefit to rural and/or remote populations. Specialist health services are often lacking in such locations or services may provide only limited resources and intermittent access. It is also likely that these locations do not provide opportunities to access sexual health aids and other products directly. The social integration of many isolated and rural settings may also limit privacy and promote “traditional” gender and family values, which restrict opportunities for sexual expression and information seeking. It has been reported, for example, that sex workers in rural areas experience significant stigma and discrimination, which can restrict access to health services [38]. Similarly, research has examined the experience of lesbian and gay people living in rural locations, noting the often unique experience of stigma, alienation and structural difficulties they encounter, restricting access to services and support [39,40]. In addition, a number of HIV-related studies have found high levels of internet use by gay men for dating and HIV information purposes for the past couple decades [41-43]. This suggests e-health technologies will be an important adjunct in improving sexual health services to this population.

## Implementation

**Figure 1:**
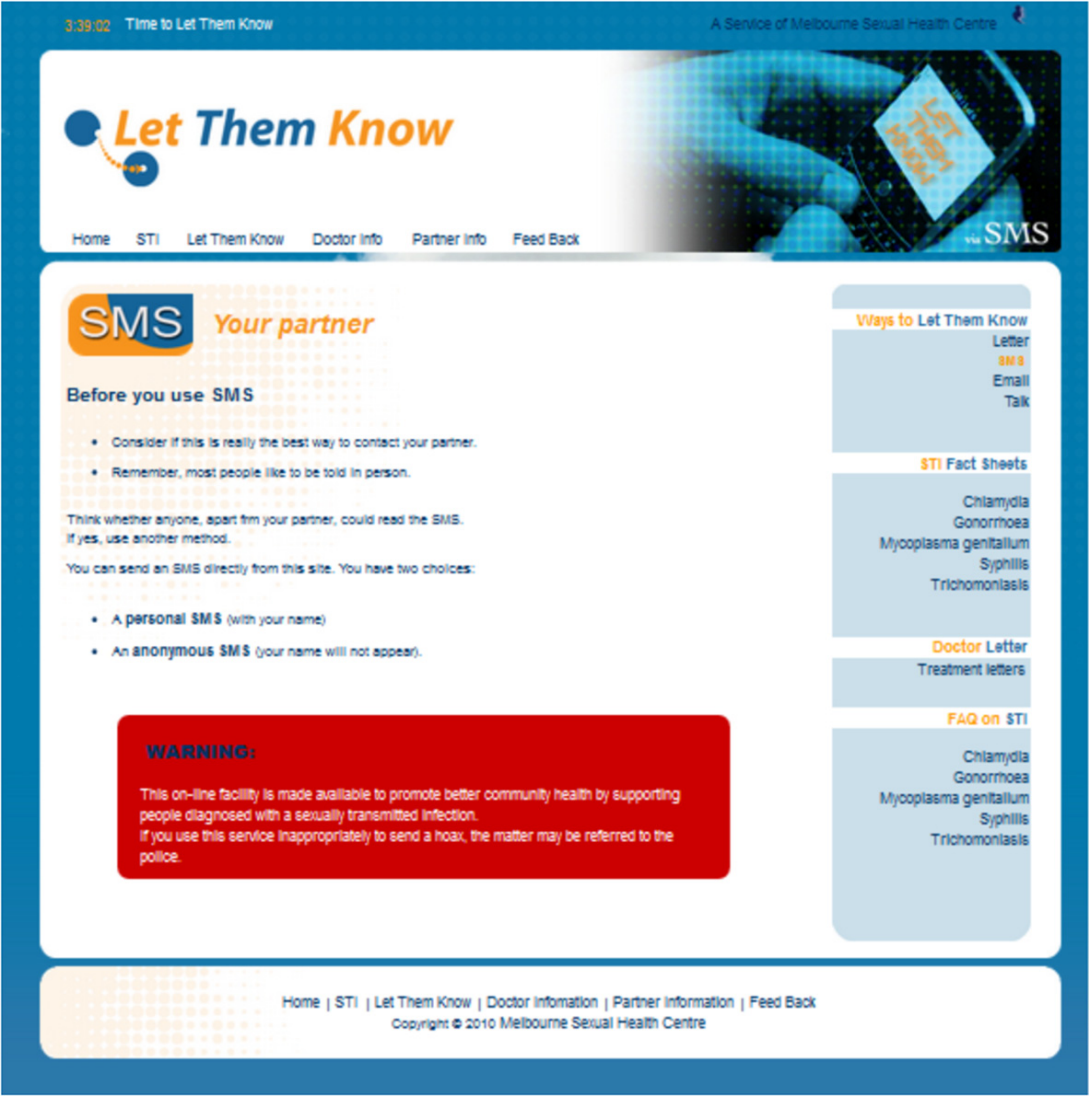
**Let Them Know website, traffic and reach from December 2008 to October 2009.** *From December 2008 to October 2009 = 6,500 visits. *From December 2008 to October 2009 = 2,700 text messages sent and 100 emails delivered.

This dimension of e-sexual health discusses the current status of online sexual health service delivery. Sexual health is one of the common health topics which young people explore on the internet and they prefer sexual health education to be interactive and anonymous [11,44]. This is a preference that SNS and text messaging can readily offer. For instance, in Australia studies have reported that sexual health based text messages were able to engage young people in the sensitive and taboo subject of sexual health [45]. In addition, text messaging and email were found to be low cost, popular and convenient mediums for sexual health promotion [46]. For example, the “Let Them Know” website service developed by the Melbourne Sexual Health Centre (http://www.letthemknow.org.au) enables people to email or text sexual partners (anonymously if desired) a notification for them to be tested for STIs. The website also provides tips on how to discuss STI infection with partners, STI factsheets, and a webpage for partners who have received a notification to get tested (see Figure 1). A similar service in the USA, “InSPOT” (http://www.inspot.org), receives over 750 visits daily, and since its inception in 2008 over 30,000 people have sent notifications for STI testing to nearly 50,000 sexual partners. By 2011, the service had expanded to many cities in the USA and Canada. Notifications sent via email include cheeky phrases such as “I got screwed while screwing, you might have too”, “It’s not what you brought to the party, it’s what you left with”, or a simple “I’m so sorry”.

Research studies consistently report that older individuals also seek information online and are one of the fastest growing groups of internet surfers who use the net to engage their sexual identities and experiences [47-50]. For older people who have access to the internet, e-health may assist in resolving restricted access to healthcare services that may result from limited physical mobility. This medium also provides a means to avoid discrimination based on physical appearance, such as wrinkles, gray hair, skin colour, and body size, and the initial prejudices older people might otherwise encounter with health professionals, peers or other community members. Online education may also reduce this phenomenon. For instance, a Massive Open Online Course (MOOC) created by the University of New England entitled “Sexuality-Based Prejudice and Discrimination” features important materials on issues of sexuality, sexual orientation, gender identity, intersex status, prejudice and queer pride. The course can be accessed for free at: http://www.eduone.net.au/module/sexually-based-prejudice-and-discrimination/. The issue of prejudice is equally applicable to gay, lesbian, bisexual, transgender, intersex and queer population groups who are vulnerable to stigmatisation and discrimination from health professionals and community members alike [51]. As such, the consumer adoption of e-health services can provide a level of anonymity that might allow these populations access to important information not sought elsewhere.

## Maintenance

In e-sexual health special considerations in regards to service delivery practices and policies are central to maintaining and improving its accessibility and integrity. Some health care consumers are attracted to the internet because they believe that it offers them anonymity in their quest to share or seek information. Considering this expectation privacy and confidentiality must not be compromised when seeking and availing e-sexual health services. Goldman and Hudson note that 80% of participants in a USA survey indicated that their willingness to engage with online health services depended on the service’s privacy policy [52]. However, many online service models depend on identifying and tracking users for a variety of purposes and often without their consent and knowledge [52]. This threat to privacy may leave consumers resistant to disclosing personal information honestly, thereby reducing online help-seeking. Until the privacy issue is treated as an essential element in the provision of online health services, e-health may remain underutilised and consumers will remain unsure of how to address their sexual health concerns adequately and confidentially.

Considering that the internet offers services to its consumers at any time of the day, it is a medium that never closes allowing consumers to gain access to websites on sexual health whenever and wherever they want. Although many internet service providers charge a monthly fee, it remains relatively affordable. Thus, e-sexual health may be an affordable and accessible resource for those on a tight budget or who live in remote geographical locations [53]. For those with less money to spare, internet access is often available for free at libraries and/or community centres, although there are issues of privacy in such public spaces. However, the provision of free sexual health information as well as links to affordable and local health care providers may be the necessary catalyst for a formal visit. In addition, due to the belief in its anonymity, the internet often offers a sense of freedom to create or change identity, reveal one’s identity as well as share intimate ideas, feelings and secrets [54,55]. This is particularly relevant for the use of e-sexual health amongst gay, lesbian, bisexual, transgender, intersex and queer (GLBTIQ) populations whose socio-sexual identities may not conform to mainstream representations.

To increase and maintain public trust and confidence in such services, policy makers would need to act by enforcing ethical information practices and ensuring health internet regulation, legislation and law. Health organization policies and procedures are needed to guarantee the privacy and integrity of online sexual health services; this would include data security and attention to ethical issues pertaining to e-health systems.

## Future directions

The provision of sexual health services can be enhanced through e-health technology. Doing this can empower consumers to engage with information technology to enhance their sexual health knowledge and quality of life. In addition, e-sexual health would support the relationship between consumers and their health care providers. Notably, a systematic and focused approach to research and the application of findings in policy and practice is required to ensure that e-health benefits all population groups.

E-health initiatives have significantly improved access to health information in rural and isolated communities. However, further research is required to assess whether barriers to e-health in rural and isolated communities, such as lack of resources, different education levels, and other socio-economic and cultural issues are negatively impacting on the implementation and utilisation of e-health-delivered sexual health services.

E-health initiatives can address the challenges of providing efficient, accessible and cost-effective sexual health care for older populations. Considering the more liberal sexual attitudes and increasing incidence of STIs within this population [56], e-sexual health programs should be targeted to ageing populations. As such, e-sexual health platforms should be evaluated not only for accessibility and financial viability but also for opportunities to integrate outreach and preventative sexual health care within local and regional health and aged care organizations.

The majority of sexual health websites have been specifically created to target young people. Most of these websites offer sexual health information about preventing unplanned pregnancy or contracting STIs. An innovative addition to such websites would be discussions and tips on how to build relationships, sexual intimacy and understanding the mutual desires of both opposite- and same-sex couples. In doing so, the e-sites could better address and combat exclusionary social behaviours (i.e., sexism, homophobia and lack of respect for others). Furthermore, young people must be taught to think critically about the information online in order for them to find accurate, comprehensive and reliable sexual health information from credible sources.

## Competing interests

The authors declare that they have no competing interests.

## Authors’ contribution

VM contributed to the conceptualization and the writing of the paper. SR and TD conducted the literature review and contributed to the writing. JS and GD contributed to the writing of the paper. All authors read and approved the final submitted paper.

## Pre-publication history

The pre-publication history for this paper can be accessed here:

http://www.biomedcentral.com/1471-2458/13/790/prepub

## References

[B1] Global eHealthhttp://www.rockefellerfoundation.org/our-work/current-work/transforming-health-systems/global-ehealth

[B2] Eysenbach G What is e-health? J Med Internet Res 2001 3 2 E20 10.2196/jmir.3.2.e20 11720962 PMC1761894

[B3] Lee RD Conley DA Preikschat A Wit capital E-health 2000 report: healthcare and the internet in the New millennium 2000 New York: Wit Capital

[B4] Savas S Parekh M Fisher L Health-e Opportunities in eHealth 1999 New York: Goldman Sachs Investment Research, The Goldman Sachs Group Inc

[B5] Hoffman DL Novak TP Venkatesh A Has the Internet become indispensable? Commun ACM 2004 47 7 37 42 10.1145/1005817.1005818

[B6] Chai SJ Aumakhan B Barnes M Jett-Goheen M Quinn N Agreda P Whittle P Hogan T Jenkins WD Rietmeijer CA Gaydos CA Internet-based screening for sexually transmitted infections to reach nonclinic populations in the community: risk factors for infection in men Sex Transm Dis 2010 37 12 756 763 10.1097/OLQ.0b013e3181e3d771 20644498 PMC3187615

[B7] Jenkins WD Rabins C Barnes M Agreda P Gaydos C Use of the internet and self-collected samples as a sexually transmissible infection intervention in rural Illinois communities Sex Health 2011 8 1 79 85 10.1071/SH10012 21371388

[B8] Rietmeijer CA Westergaard B Mickiewicz TA Richardson D Ling S Sapp T Jordan R Wilmoth R Kachur R McFarlane M Evaluation of an online partner notification program Sex Transm Dis 2011 38 5 359 364 10.1097/OLQ.0b013e31820ef796 21343844

[B9] Shoveller J Knight R Davis W Gilbert M Ogilvie G Online sexual health services: examining youth’s perspectives Can J Public Health 2011 103 1 14 18 22338322 10.1007/BF03404062 PMC6973649

[B10] Gray NJ Klein JD Cantrill JA Noyce PR Adolescent girls’ use of the internet for health information: issues beyond access J Med Syst 2002 26 6 545 553 10.1023/A:1020296710179 12385536

[B11] Buhi ER Daley EM Fuhrmann HJ Smith SA An observational study of how young people search for online sexual health information J Am Coll Health 2009 58 2 101 111 10.1080/07448480903221236 19892646

[B12] Fox S Online health search 2006 2006 WashingtonDC: Pew Internet & American Life Project

[B13] Ackard DM Neumark-Sztainer D Health care information sources for adolescents: age and gender differences on use, concerns, and needs J Adolesc Health 2001 29 3 170 176 10.1016/S1054-139X(01)00253-1 11524215

[B14] Glasgow RE Vogt TM Boles SM Evaluating the public health impact of health promotion interventions: the RE-AIM framework Am J Public Health 1999 89 1322 1327 10.2105/AJPH.89.9.1322 10474547 PMC1508772

[B15] Glasgow RE EHealth evaluation and dissemination research Am J Prev Med 2007 32 S119 S126 10.1016/j.amepre.2007.01.023 17466816

[B16] Carpenter KM Stoner SA Mikko AN Dhanak LP Parsons JT Efficacy of a web-based intervention to reduce sexual risk in men who have sex with men AIDS Behav 2010 14 3 549 557 10.1007/s10461-009-9578-2 19499321 PMC3128504

[B17] Noar SM Black HG Pierce LB Efficacy of computer technology-based HIV prevention interventions: a meta-analysis AIDS 2009 23 1 107 115 10.1097/QAD.0b013e32831c5500 19050392

[B18] Internet world stathttp://www.internetworldstats.com/stats.htm

[B19] Fox S The social life of health information 2011 Washington DC: Pew Internet & American Life Project

[B20] Fox S Rainie L Vital decisions: how internet users decide what information to trust when they 2002 Washington DC: Pew Internet & American Life Project

[B21] Madden M Rainie L America’s online pursuits: the changing picture of who’s online and what they do 2003 Washington DC: Pew Internet & American Life Project

[B22] Eysenbach G Diepgen TL The role of e-health and consumer health informatics for evidence-based patient choice in the 21st century Clin Dermatol 2001 19 1 11 17 10.1016/S0738-081X(00)00202-9 11369478

[B23] Eysenbach G Recent advances: consumer health informatics BMJ 2000 320 7251 1713 10.1136/bmj.320.7251.1713 10864552 PMC1127483

[B24] Bennett J The worldwide web and sexual health 2011 http://www.thefreelibrary.com/The-worldwide-web-and-sexual-health-a0284553417

[B25] Powell J Darvell M Gray J The doctor, the patient and the world-wide web: how the internet is changing healthcare JRSM 2003 96 2 74 76 10.1258/jrsm.96.2.74 PMC539397 12562977

[B26] Gold J Lim M Hellard M Hocking J Keogh L What’s In a message? delivering sexual health promotion to young people in Australia via text messaging BMC Publ Health 2010 10 1 792 10.1186/1471-2458-10-792 PMC3022861 21190584

[B27] Cline RKW Haynes KM Consumer health information seeking on the internet: the state of the art Health Edu Res 2001 16 6 671 692 10.1093/her/16.6.671 11780707

[B28] Health e Technologie initiative Using eHealth interventions to engage consumers: a practical guide 2008 Boston: Health e Technologie initiative, Brigham and Women’s Hospital

[B29] Li CP A tentative discussion of the limitations of health information on the internet in Taiwan AJHIS 2007 2 1–4 103 115

[B30] Kreps GL Neuhauser L New directions in eHealth communication: opportunities and challenges Patient Educ and Couns 2010 78 3 329 336 10.1016/j.pec.2010.01.013 20202779

[B31] Viswanath K Kreuter MW Health disparities, communication inequalities, and e-health: a commentary Am J Prev Med 2007 32 Suppl 5 131 133 17466818 10.1016/j.amepre.2007.02.012 PMC2043145

[B32] Baur C Kanaan SB Expanding the reach and impact of consumer e-health tools 2006 Rockville: Office of Disease Prevention and Health Promotion, US Department of Health and Human Services

[B33] Maloney S Ilic D Green S Accessibility, nature and quality of health information on the internet: a survey on osteoarthritis Rheumatology 2005 44 3 382 385 10.1093/rheumatology/keh498 15572390

[B34] Dutta-Bergman MJ The impact of completeness and Web Use motivation on the credibility of eHealth information J Commun 2004 54 2 253 269 10.1111/j.1460-2466.2004.tb02627.x

[B35] Harrison JP Lee A The role of e-health in the changing health care environment Nurs Econ 2006 24 6 283 288 17266004

[B36] Appleby C Health care. com. Trustees may soon find themselves on the board of an internet startup Trustee 2000 53 1 18 11785212

[B37] Kwankam SY What e-health can offer Bull World Health Organ 2004 82 10 800 802 15643805 PMC2623036

[B38] Scott J MacPhail C Minichiello V Bang and bust: almost everything you wanted to know about sex and the mining boom (but were afraid to ask) Preview: Australian Society of Exploration Geophysicists 2012 160 26 31

[B39] Bell D Valentine G Queer country: rural lesbian and gay lives J Rural Stud 1995 11 2 113 122 10.1016/0743-0167(95)00013-D

[B40] Preston DB D’Augelli AR Kassab CD Starks MT The relationship of stigma to the sexual risk behavior of rural men who have sex with men AIDS Educ Prev 2007 19 3 218 230 10.1521/aeap.2007.19.3.218 17563276

[B41] Benotsch E Kalichman S Cage M Men who have met sex partners via the internet: prevalence, predictors and implications for HIV prevention Arch Sex Behav 2002 31 2 177 183 10.1023/A:1014739203657 11974643

[B42] Hospers HJ Harterink P van den Hoek K Veenstra JD Chatters on the internet: a special target group for HIV prevention AIDS Care 2002 14 4 539 544 12204155 10.1080/09540120208629671

[B43] Ross MW The internet as a medium for HIV prevention and counselling Focus 2002 17 5 4 6 12064293

[B44] Baxter L Egbert N Ho E Everyday health communication experiences of college students J of ACH 2008 56 4 427 436 18316288 10.3200/JACH.56.44.427-436

[B45] Bailey JV Murray E Rait G Mercer CH Morris RW Peacock R Cassell J Nazareth I Interactive computer-based interventions for sexual health promotion Cochrane Database Syst Rev 2010 9 CD006483 10.1002/14651858.CD006483.pub2 PMC13139710 20824850

[B46] Lim MS Hocking JS Aitken CK Impact of text and email messaging on the sexual health of young people: a randomised controlled trial J Epidemiol Community Health 2012 66 1 69 74 10.1136/jech.2009.100396 21415232

[B47] Campbell RJ Consumer informatics: elderly persons and the internet Perspect Health Inf Manag 2005 2 2 1 16 18066370 PMC2047318

[B48] Leung A Ko P Chan KS Chi I Chow NWS Searching health information via the web: Hong Kong Chinese older adults’ experience Public Health Nurs 2007 24 2 169 175 10.1111/j.1525-1446.2007.00621.x 17319889

[B49] Flynn KE Smith MA Freese J When do older adults turn to the internet for health information? findings from the Wisconsin longitudinal study J Gen Intern Med 2006 21 12 1295 1301 10.1111/j.1525-1497.2006.00622.x 16995892 PMC1924748

[B50] Cutilli CC Seeking health information: what sources do your patients use? Orthop Nurs 2010 29 3 214 219 20505493 10.1097/NOR.0b013e3181db5471

[B51] Myer I Northridge ME The health of sexual minorities: public health perspectives on lesbian, gay, bisexual and transgender populations 2007 New York: Columbia University Press

[B52] Goldman J Hudson Z Virtually exposed: privacy and e-health Health Aff 2000 19 6 140 148 10.1377/hlthaff.19.6.140 11192397

[B53] Adams M Oye J Parker T Sexuality of older adults and the internet: from sex education to cybersex Sex Relation Ther 2003 18 3 405 415 10.1080/1468199031000153991

[B54] Cooper A Boies S Maheu M Greenfield D Szuchman LT, Muscarella F Sexuality and the internet: the next sexual revolution Psychological perspectives on human sexuality 2001 New York: John Wiley & Sons 519 545

[B55] Waskul D Douglass M Edgley C Cybersex: outercourse and the enselfment of the body Symb Interaction 2000 23 4 375 397 10.1525/si.2000.23.4.375

[B56] Minichiello V Rahman S Hawkes G Pitts M STI epidemiology in the global older population: emerging challenges Perspect Public Health 2012 132 4 178 181 10.1177/1757913912445688 22729008

